# High quality monocular depth estimation with parallel decoder

**DOI:** 10.1038/s41598-022-20909-x

**Published:** 2022-10-05

**Authors:** Jiatao Liu, Yaping Zhang

**Affiliations:** School of Information Science and Technology, Yunnan Normal University, Kunming, 650500 Yunnan China

**Keywords:** Applied optics, Optical techniques

## Abstract

Monocular depth estimation aims to recover the depth information in three-dimensional (3D) space from a single image efficiently, but it is an ill-posed problem. Recently, Transformer-based architectures have achieved excellent accuracy in monocular depth estimation. However, due to the characteristics of Transformer, the model parameters are huge and the inference speed is slow. In traditional convolutional neural network–based architectures, many encoder-decoders perform serial fusion of the multi-scale features of each stage of the encoder and then output predictions. However, in these approaches it may be difficult to recover the spatial information lost by the encoder during pooling and convolution. To enhance this serial structure, we propose a structure from the decoder perspective, which first predicts global and local depth information in parallel and then fuses them. Results show that this structure is an effective improvement over traditional methods and has accuracy comparable with that of state-of-the-art methods in both indoor and outdoor scenes, but with fewer parameters and computations. Moreover, results of ablation studies verify the effectiveness of the proposed decoder.

## Introduction

Depth estimation from two-dimensional (2D) RGB images has a wide range of applications, such as 3D reconstruction, scene understanding, automatic driving, and robotics. Depth estimation can usually be divided into monocular depth estimation and binocular depth estimation according to the number of cameras used to capture images. Monocular depth estimation obtains a 3D scene from a single image and is suitable for applications with only one view of the scene. However, since a single image corresponds to an infinite number of 3D scenes, it is still challenging to estimate the depth through one picture^1^.

In the 2020s, with the emergence of large-scale datasets and the improvement of hardware computing capabilities, Vision Transformer (ViT) outperformed convolutional models for the first time in recognition tasks^2^. Then, a ViT-based architecture was applied to the monocular depth estimation task and achieved excellent accuracy performance^3^. In the work, the ViT encoder based on transfer learning and the convolutional network decoder based on recombination fusion are used as the two major parts of the deep neural network. However, the ViT-based models have high hardware requirements because the ViT structure contains a huge number of parameters and the calculation process is complicated. For traditional convolutional neural network (CNN)–based architectures, many researchers have designed highly accurate deep-learning network architectures by combining global overall information and local detailed information, and many of these architectures achieved prediction through the serial connection from global to local prediction. The general idea presented in this work is to design an architecture for parallel prediction of global and local depth information based on the encoder-decoder network architecture, and to use a learnable module to integrate these predictions.

A CNN, when used as the backbone of a depth estimation network, may lose the fine-grained information of the input image in the deeper stages since it uses explicit downsampling and convolution operations to gradually expand the receptive field. Therefore, skip connections from multiple stages of the encoder to the decoder are added to many architectures appropriately^4^. From the perspective of skip connections, we directly decode the multi-stage and multi-resolution features to obtain multi-stage prediction. Moreover, we accordingly design a loss term that allows the parallel prediction of each stage to have the depth value range of its concern.

The main contributions of this paper are the following: (1) we propose a novel end-to-end parallel depth estimation architecture based on CNNs, which shows significant improvement over traditional methods and achieves state-of-the-art performance; (2) we correspondingly define a loss term based on the depth interval division; (3) we design an efficient and lightweight decoder that can be applied to more backbone networks.

## Related work

Monocular depth estimation from a single RGB image is an ill-posed problem since one picture can correspond to an infinite number of 3D scenes. Additionally, problems such as lack of scene coverage and semi-transparent or reflective materials can lead to fuzzy situations in which geometry cannot be derived from the appearance. In the past, methods that rely on CNNs can generate high-quality depth maps from a single RGB input image. CNN is a feedforward neural network that includes convolution computation^5^. Compared with general neural networks, CNNs have the advantages that the network structure can better adapt to the image structure, and weight sharing can reduce the parameters of the network^6^.

Monocular depth estimation is a low-cost method of distance measurement. When a supervised method is used to train the neural network, the supervised labels are usually obtained by a distance sensor such as an RGB-D camera or a multi-channel laser scanner. The method of using deep learning for monocular depth estimation started from a two-scale network proposed by Eigen et al.^1^. Then, several researchers proposed many efficient methods based on deep learning that use CNNs. Laina et al.^7^ used a fully convolutional residual network based on ResNet-50 and replaced the fully connected layer with a series of upsampling blocks. Alhashim et al.^8^ introduced skip connections in a simple encoder-decoder network architecture, and trained the model using transfer learning. Lee et al.^9^ replaced the standard upsampling layers with the local planar guidance layers to guide the features to full resolution during decoding. Long et al.^10^ proposed an adaptive surface normal constraint method that effectively correlates the depth estimation with geometric consistency, which can faithfully reconstruct 3D geometry and is robust to local shape changes. Yin et al.^11^ designed a loss term to enforce a simple geometric constraint, which can greatly improve the accuracy of depth estimation. Chen et al.^12^ proposed a structure-aware residual pyramid network to exploit multi-scale structures in complex scenes for accurate depth prediction. Fu et al.^13^ found that if the depth regression task is transformed into a classification task its performance can be improved. Bhat et al.^14^ designed the AdaBins module, which divides the depth range into 256 intervals, with the center value of each interval being the depth value of the pixels falling in the interval, and the final depth of a pixel being a linear combination of the center depth values of the intervals.

Transfer learning is attracting increasingly more attention since it can make full use of the previously marked data while ensuring accuracy of the model on the new task. A recent transfer learning method has shown its effectiveness in many tasks, such as image classification^15,16^ and image semantic segmentation^17^. For monocular depth estimation tasks, several researchers have used a pre-trained model with high accuracy in image-classification tasks as the encoder in the network architecture^8,9,14^.

Transformer, a self-attention-based architecture, has been widely used in natural language processing (NLP) in recent years^18,19^. Dosovitskiy et al.^2^ applied Transformer architecture from NLP to image classification directly, and the model is particularly successful when it is instantiated as a high-capacity architecture and trained on a very large dataset. Ranftl et al.^3^ applied this ViT to monocular depth estimation, and also obtained a high-precision depth estimation model through training on a large number of datasets.

## Proposed method

In this section, we detail our method for depth estimation from a single image. Our main idea is to design an end-to-end deep-learning network architecture that can predict the global and local depth information of the input image in parallel. The overall architecture of the proposed model follows encoder-decoder network architecture, and the way to combine local and global information is to use the Transformer encoder, which is able to achieve global self-attention of the image.

### Motivation

Even though ViT-based architectures have achieved excellent performance, their problems, including the huge number of parameters and slow inference speed, cannot be ignored; in addition, the model requires a large quantity of data to train. These problems may be acceptable for tasks with low-resolution inputs, but quickly become intractable for higher-resolution inputs. In the depth estimation task, the input image usually has a higher resolution, and the use of CNN-based architecture may have more application significance.

In traditional CNN-based architectures, for the ill-posed problem of monocular depth estimation, many researchers try to explore solutions by synthesizing global and local depth information. The local details of the input image may be lost in CNN-based encoders since they use gradual downsampling to gradually increase the receptive field. We propose to use CNNs to design neural networks in a way that does not lose detailed information as much as possible.

The shallower features of an encoder have higher resolution and contain more details and spatial information. Therefore, we propose to calculate these high-resolution features directly to obtain predictions with rich local details and fuse them with more accurate global predictions obtained from deeper parts of the neural network. Many prior works simply upsampled the deep features and progressively concatenated them with the shallower features. It may be difficult in this method to recover the spatial information lost by the encoder during pooling and convolution, which causes boundary blur in high-resolution predictions. Therefore, we propose a parallel decoder structure that directly uses the multi-resolution feature vectors of the encoder in multiple stages for parallel prediction, and then fuses the multi-stage predictions for output.

### Network architecture

**Figure 1:**
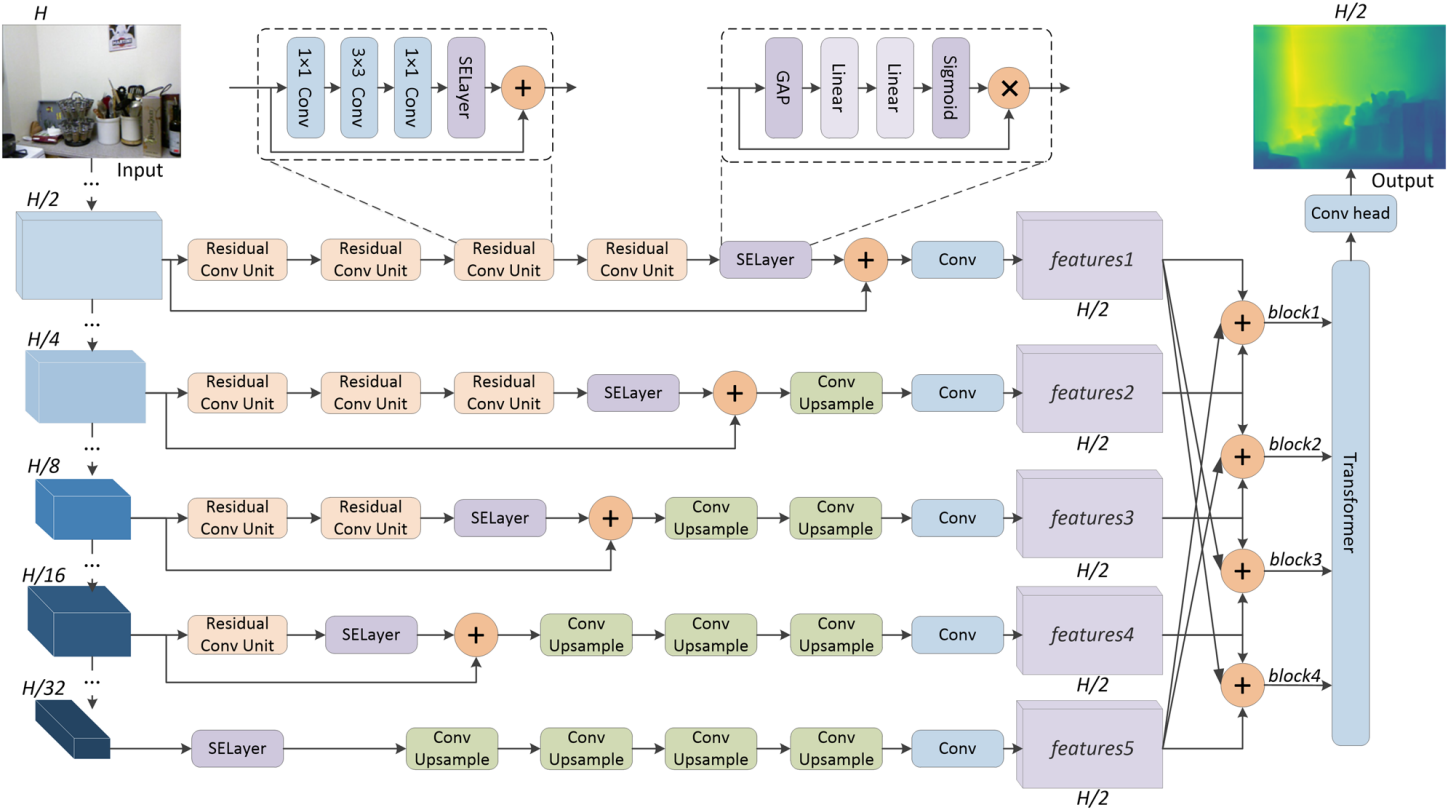
Structure of the proposed model. The structure follows encoder-decoder network architecture; the encoder uses the backbone network in the image classification task via transfer learning; the decoder obtains the output by performing parallel computation on the features encoded in the five stages of the encoder and then fusing them; and the output is half the spatial resolution of the input.

Figure 1 shows the overview of the proposed network architecture for depth estimation. For the encoder, we use EfficientNet-B7^20^ pre-trained on ImageNet^21^. The input image is encoded into multi-scale feature vectors at different stages of the encoder. The different feature vectors first go through a series of SENet-based residual blocks proposed by Hu et al.^22^, which consists of three convolution layers and a SELayer. SELayer is a channel attention mechanism. It first performs the average pooling operation to shrink feature maps through spatial dimensions, and then calculates the weight of each channel through two fully connected layers and a sigmoid operation, and multiplies them with the corresponding channel features, respectively, to reweight the feature maps. After that, the feature vectors perform a series of convolution and resampling operations to obtain features with the same shape as shown in *features1* to *features5* in Fig. 1, and perform feature fusion to obtain parallel prediction results that focus on global or local as shown in *block1* to *block4* in Fig. 1. Finally, self-attention-based Transformer is used to fuse the predictions, and the final prediction is output through the convolution head.

Specifically, for the encoder, we choose features with post-encoding resolutions of 1/2, 1/4, 1/8, 1/16, and 1/32 of the input resolution. For the decoder, features with higher resolution go through more SENet-based residual blocks. Note that we add a separate channel attention layer after the last residual block and add a residual connection from the encoder to this layer. The feature maps with different scales are convolved and resampled to have the same number of channels and resolution. Considering the memory limit of the graphical processing unit (GPU) employed, we set the number of the channels to 30 and the resolution to 1/2 of the input resolution. The shallower features contain more local details and spatial information, and the deeper features contain more accurate predictions of global depth information. For *block1* to *block4*, we hope that deeper predictions will focus more on global predictions. The local predictions need to refer to the global predictions, and the reverse is also true. Therefore, as shown in Fig. 1, *block1* is obtained by convolution of *features1*, *features2*, and *features5*; *block2* is obtained by convolution of *features2*, *features3*, and *features5*; *block3* is obtained by convolution of *features3*, *features4*, and *features1*; *block4* is obtained by convolution of *features4*, *features5*, and *features1*.

**Figure 2:**
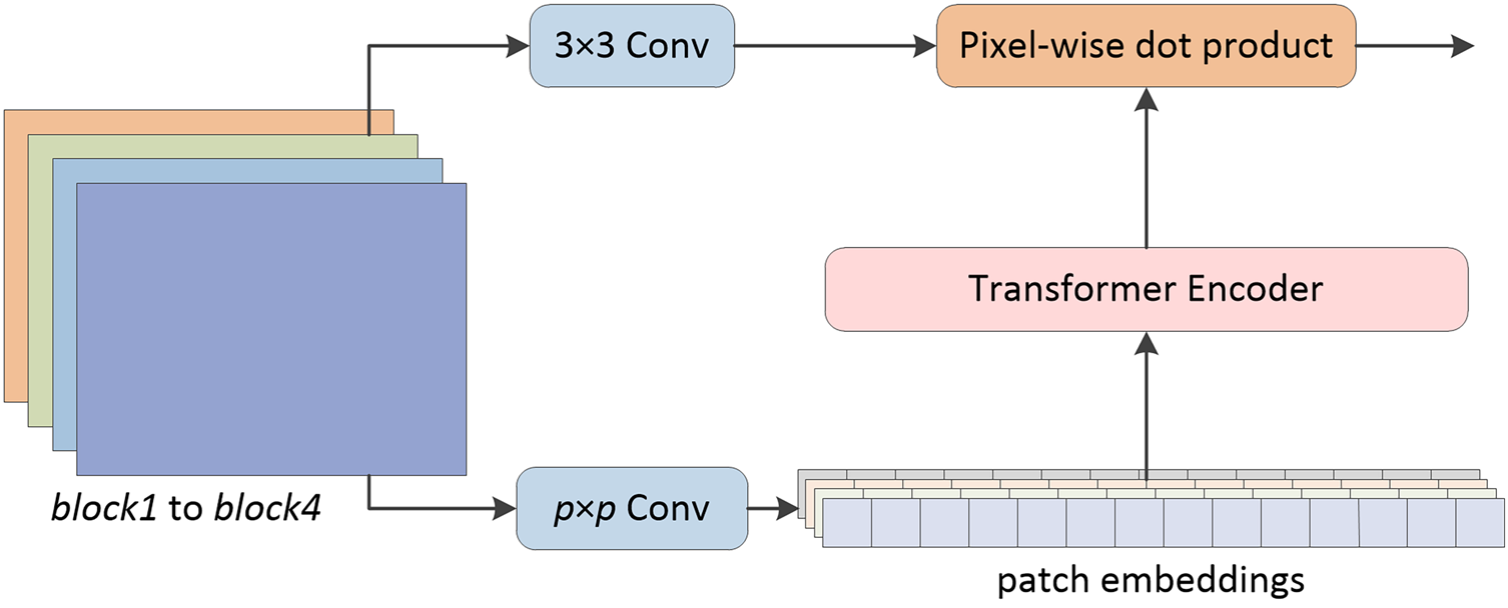
Structure of the fusion block.

The Transformer module that fuses the predictions of *block1* to *block4* can be regarded as a simplified version of Adabins proposed by Bhat et al.^14^. As shown in Fig. 2, we must convert these 2D tensors into 1D sequences before inputting the predictions into Transformer. The process is as follows:

1 $$x \in {\mathbb{R}}^{{\frac{H}{2} \times \frac{W}{2} \times 4}} \to x \in {\mathbb{R}}^{{\frac{H}{2p} \times \frac{W}{2p} \times E}} \to x \in {\mathbb{R}}^{S \times E} .$$

Specifically, we first pass these predictions through a convolutional layer with kernel size $p \times p$, stride *p*, and output channels *E*. Thus, the output of this convolution is a tensor of shape $\frac{H}{2p} \times \frac{W}{2p} \times E$; we then flatten this tensor to $S \times E$, where $S = \frac{HW}{{4p^{2} }}$. In this way, a set of feature vectors that can be input into the Transformer encoder is obtained. After restoring the output of the Transformer encoder as a 2D tensor, we use it as the weight matrix ${\mathbb{N}}$. On the other side, we pass the predictions through a convolutional layer with kernel size $3 \times 3$ and obtain a tensor ${\mathbb{Z}}$ of shape $\frac{H}{2} \times \frac{W}{2} \times C$. After doing the pixel-wise dot product of ${\mathbb{N}}$ and ${\mathbb{Z}}$, the final predicted depth map is output through the convolution head. In our experiment, *p* was set to 16, *E* to 4, and *C* to 128.

### Loss function

*Pixel-level depth loss for block1 to block4*. We first introduced the loss function for *block1* to *block4* as shown in Fig. 1. Assuming that the maximum depth in the ground truth is $d\_max$ and the minimum depth is $d\_min$, we first divided the depth range [$d\_min$, $d\_max$] into *b* intervals on average; that is, the length of a single interval is $len = \frac{d\_max - d\_min}{b}$, and the depth range corresponding to the *i*th ($i \le b$) interval is [$d\_min + \left( {i - 1} \right) \times len$, $d\_min + i \times len$]. Then we made a histogram of the ground truth in these *b* intervals to find the interval that accounts for the largest proportion of the depth of the scene; most of the global information is contained in this interval. Correspondingly, the interval that accounts for the smaller proportion contains more local information. In our experiment, *b* was set to 10, and we arranged the 10 intervals in descending proportions. Note that most of the depth values in a scene are distributed in the first and second intervals, which is unfavorable for optimizing the model in parallel, so we proposed that *block1* focuses on the fifth to tenth interval, *block2* on the fourth to eighth interval, *block3* on the second to fourth interval, and *block4* on the first and second interval. We then calculated the mean-squared-error loss on these intervals separately:

2 $$L_{focus} \left( {y,block1,block2,block3,block4} \right) = \mathop \sum \limits_{i = 1}^{4} \left( {\frac{{\lambda_{i} }}{{n_{i} }}\mathop \sum \limits_{{p_{i} }}^{{n_{i} }} d_{{p_{i} }}^{2} } \right),$$

where *y* is the ground truth, $\lambda_{1} = 0.5$, $\lambda_{2} = \lambda_{3} = 0.6$, $\lambda_{4} = 1$, $n_{i}$ is the total number of pixels after *y* passes through the mask of the corresponding interval, $d_{{p_{i} }} = y_{{p_{i} }} - blocki_{{p_{i} }}$, and $y_{{p_{i} }}$ and $blocki_{{p_{i} }}$ are the depth value of the pixel $p_{i}$ in the masked ground truth *y* and $blocki$, respectively.

*Pixel-level depth loss for output*. We define the pixel-level loss for the output as a combination of two parts: one is the mean-squared-error loss of each pixel depth value in log space, and the other is the variance of the error of each pixel depth value in the log space. Note that the second part is used to better predict small details in the image that are more difficult to predict accurately. To improve the sensitivity when the loss value gradually tends to zero, the loss term is a square root of the combination:

3 $$L_{depth} \left( {y,\hat{y}} \right) = \sqrt {\frac{1}{n}\mathop \sum \limits_{p} d_{p}^{2} + \frac{1}{n}\mathop \sum \limits_{q}^{n} \left( {\frac{1}{n}\mathop \sum \limits_{p}^{n} d_{p} - d_{q} } \right)^{2} } ,$$

where $d_{p} = {\text{ln}}y_{p} - {\text{ln}}\hat{y}_{p}$, $y_{p}$ and $\hat{y}_{p}$ are the depth values of the pixel *p* in the ground truth *y* and the predicted depth map $\hat{y}$, respectively, and *n* is the total number of pixels in the depth map. Therefore, this loss term can not only control the global overall prediction, but also stabilize the local detailed prediction.

*Multi-scale structure similarity loss for output.* The multi-sce structure similarity loss (MS-SSIM) loss function scales the picture from large to small according to certain rules, and then calculates the structural similarity, which is equivalent to considering the resolution of the image and retaining the high-frequency information in the image^23^. This loss term is defined as follows:

4 $$L_{{ms{ - }ssim}} \left( {y,\hat{y}} \right) = \sqrt {1 - {\text{MS - SSIM}}\left( {y,\hat{y}} \right)} .$$

The training loss is defined as follows:

5 $$L = \eta L_{focus} + \alpha L_{depth} + \beta L_{{ms{ - }ssim}} ,$$

where $$\eta = 0.1$$, $$\alpha = 8$$, and $$\beta = 10$$.

## Experiments

We conducted an extensive set of experiments to verify the effectiveness of our method. First, we describe the datasets used and then present the implementation details of the proposed method and evaluate it qualitatively and quantitatively. Finally, we carried out a series of ablation studies to facilitate discussion of the core factors proposed in detail.

### Datasets

NYU Depth v2 is a commonly used dataset in depth estimation tasks. It provides RGB images of different indoor scenes and their corresponding depth maps. The image depth ranges from 0 to 10 m and the image resolution is 480 × 640^24^. The dataset contains approximately 120,000 training samples, and we trained our model on the subset of 50,688 samples supplied by Alhashim et al.^8^. The depth values of some pixels in the original depth map in the dataset are missing due to the hardware of the acquisition device. The dataset uses the method proposed by Levin et al.^25^ to fill in the missing parts. In addition, we use image random horizontal flip and random color to expand the training data to reduce overfitting and improve the generalization performance of the model. Random color augmentation includes randomly changing the saturation, hue, brightness, gamma and contrast of an image. The resolution of the final output depth map is half of the input image. For evaluation, we use the 654 official testing images as the input of the model, and the output is upsampled by a factor of 2 to match the original depth resolution. In addition, the cropping boundary method proposed by Eigen et al.^1^ is used. After the original image and horizontally flipped image are used as the model input, the average of the predicted depth of the two images is taken as the final output of the model and evaluated.

KITTI provides outdoor stereo images and corresponding 3D laser scanning, which are captured by equipment mounted on a moving vehicle^26^. It includes 61 scenes collected from “city”, “residential”, “road” and “campus” categories, with an image resolution of 1241 × 376, and provides sparse ground truth. For training, the images are randomly cropped to 704 × 352, and the data augmentation of random horizontal flipping and random color is used. For testing, we use 652 test images out of 697 test images split by Eigen et al.^1^ to evaluate the model performance (45 images without corresponding ground truth are excluded from the test set). The central cropping scheme proposed by Grag et al.^27^ is also adopted for the evaluation. We bilinearly upsample the model prediction to match the ground truth resolution. The final depth is computed in the same way as for NYU Depth V2 dataset.

SUN RGB-D is an indoor dataset that has more diverse scenes and contains approximately 10,000 images collected by four different sensors^28–30^. For our experiments only the official test set of 5,050 images were used for testing, without training.

DIODE (Dense Indoor/Outdoor DEpth) is a dataset that contains thousands of diverse high resolution color images with accurate, dense, long-range depth measurements^31^. We use the validation set for testing, which contains 325 indoor images and 446 outdoor images.

ETH-3D provides multi-view stereo/3D reconstruction benchmark covering various indoor and outdoor scenes^32^. Ground truth geometry has been obtained by using a high-precision laser scanner. In the training set with ground truth, 219 indoor images in 7 scenes and 235 outdoor images in 6 scenes are used for our cross-dataset testing.

ScanNet is an indoor RGB-D video dataset, containing 2.5 million views in more than 1500 scans, annotated with 3D camera poses, surface reconstructions, and instance-level semantic segmentations^33^. We use 100 test scenes, including 2135 frames, to evaluate cross-dataset generalization ability.

### Implementation details

We used the PyTorch^34^ framework to implement the proposed network structure, and trained the model on a single NVIDIA GeForce RTX 3090 GPU. When training the model, we used AdamW^35^ with a weight decay of $10^{{ - 2}}$ as the optimizer. For learning rate, we used the 1-cycle strategy^36^ with a maximum learning rate of $2 \times 10^{{{ - }4}}$. In the first 30% of iterations, the learning rate linearly warmed up from $\frac{{2 \times 10^{{{ - }4}} }}{30}$ to $2 \times 10^{{{ - }4}}$, and then cosine-annealed to $\frac{{2 \times 10^{{{ - }4}} }}{60}$. The batch size was set to 4. We trained 20 epochs on NYU Depth v2 dataset and 30 epochs on KITTI dataset, taking 105 min and 49 min per epoch respectively. The proposed model has about 77.0 × 10^6^ parameters: 63.8 × 10^6^ for the encoder and 13.2 × 10^6^ for the decoder.

### Evaluation

*Quantitative evaluation*. Table 1 shows the quantitative comparison between the proposed method and eight state-of-the-art methods on NYU Depth v2 dataset, using six standard metrics introduced by Eigen et al.^1^. These metrics are threshold accuracy ($\delta_{i} , i = {1,2,3}$), average relative error (REL), logarithmic average error (Log10), and root-mean-square error (RMS), and are defined, respectively, as follows:

6 $$\delta_{i} = {\text{max}}\left( {\frac{{y_{p} }}{{\hat{y}_{p} }},\frac{{\hat{y}_{p} }}{{y_{p} }}} \right) < thr,$$

7 $${\text{REL}} = \frac{1}{n}\mathop \sum \limits_{p}^{n} \frac{{\left| {y_{p} - \hat{y}_{p} } \right|}}{{y_{p} }},$$

8 $${\text{Log10}} = \frac{1}{n}\mathop \sum \limits_{p}^{n} \left| {{\text{log}}_{10} \left( {y_{p} } \right) - {\text{log}}_{10} \left( {\hat{y}_{p} } \right)} \right|,$$

9 $${\text{RMS}} = \sqrt {\frac{1}{n}\mathop \sum \limits_{p}^{n} \left( {y_{p} - \hat{y}_{p} } \right)^{2} } ,$$

where in Eq. (6), when $i = 1,\;2,\;3$, $thr = 1.25,\;1.25^{2} ,\;1.25^{3}$, respectively. Additionally for KITTI dataset, as shown in Table 2, we use the two standard metrics: square relative difference (Sq. Rel) and root-mean-square error in the log space (RMS log), as follows:

10 $${\text{Sq}}{\text{. Rel}} = \frac{1}{n}\mathop \sum \limits_{p}^{n} \frac{{ \left\| y_{p} - \hat{y}_{p} \right\|}}{{y_{p} }},$$

11 $${\text{RMS log}} = \sqrt {\frac{1}{n}\mathop \sum \limits_{p}^{n} \left\| \ln y_{p} - \ln \hat{y}_{p}\right\|^{2} } .$$

**Table 1:** Quantitative comparison with prediction results of other models on NYU Depth v2 dataset.

Method	δ_1_↑	δ_2_↑	δ_3_↑	REL↓	RMS↓	Log_10_↓
Eigen et al.^1^	0.769	0.950	0.988	0.158	0.641	–
Laina et al.^7^	0.811	0.953	0.988	0.127	0.573	0.055
Fu et al.^13^	0.828	0.965	0.992	0.115	0.509	0.051
Alhashim et al.^8^	0.846	0.974	0.994	0.123	0.465	0.053
Yin et al.^11^	0.875	0.976	0.994	0.108	0.416	0.048
Lee et al.^9^	0.885	0.978	0.994	0.110	0.392	0.047
Bhat et al.^14^	0.903	0.984	*0.997*	*0.103*	0.364	*0.044*
Ranftl et al.^3^	*0.904*	**0.988**	**0.998**	0.110	**0.357**	0.045
Proposed	**0.905**	*0.987*	*0.997*	**0.101**	*0.359*	**0.043**

The best results are shown in bold, and the second best results are italics.

**Table 2:** Quantitative comparison with prediction results of other models on KITTI dataset.

Method	δ_1_↑	δ_2_↑	δ_3_↑	REL↓	Sq. Rel↓	RMS↓	RMS log↓
Eigen et al.^1^	0.702	0.898	0.967	0.203	1.548	6.307	0.282
Fu et al.^13^	0.932	0.984	0.994	0.072	0.307	2.727	0.120
Alhashim et al.^8^	0.886	0.965	0.986	0.093	0.589	4.170	–
Yin et al.^11^	0.938	0.990	0.998	0.072	–	3.258	0.117
Lee et al.^9^	0.956	*0.993*	*0.998*	0.059	0.245	2.756	0.096
Bhat et al.^14^	*0.964*	**0.995**	**0.999**	*0.058*	**0.190**	**2.360**	**0.088**
Ranftl et al.^3^	0.959	**0.995**	**0.999**	0.062	0.222	2.575	*0.092*
Proposed	**0.965**	**0.995**	**0.999**	**0.057**	*0.197*	*2.368*	**0.088**

The best results are shown in bold, and the second best results are italics.

From the Tables 1 and 2, the proposed method is comparable to the state-of-the-art methods in terms of accuracy and error.

We considered the methods proposed by Bhat et al.^14^ and Ranftl et al.^3^ as the two most important competitors. Note that while the encoders of both methods and the proposed method are pre-trained on ImageNet, the method presented in Ref.^3^ needed extra training data and fine-tuning on NYU Depth v2 dataset to obtain better results. Specifically, the method in Ref.^3^ must first be trained on a dataset with 1.4 × 10^6^ images for 60 epochs and then fine-tuned on NYU Depth v2, but the model of Bhat et al.^14^ and that proposed herein only need be trained on a 50,688-image subset of NYU Depth v2 for 25 and 20 epochs, respectively. We compared the complexity of the models, including the amount of parameters, computation and the inference speed. The amount of computation of the model is measured by the MACs (Multiply-Accumulate operations) with an input resolution of 480 × 640. As shown in Table 3, the proposed model has fewer parameters and computations than the other two models, and the inference speed is the fastest among the three models. Note that our experiments for inference speed were done on a single NVIDIA GeForce GTX 1660ti GPU, and since the output resolution of the method in Ref.^3^ is twice that of the method in Ref.^14^ and the proposed method, the results from both Ref.^14^ and the proposed one also include the time for 2 × upsampling. Moreover, the time in the table is obtained by averaging the time after 5050 inferences.

**Table 3:** Model statistics.

Method	Parameters (× 10^6^)	MACs (× 10^9^)	Time (ms)	FPS
Bhat et al.^14^	78.26	186.33	108	9.26
Ranftl et al.^3^	123.15	229.11	199	5.03
Proposed	76.99	79.73	103	9.71

To further investigate the generalization ability of the proposed model, we tested the four models pre-trained on NYU Depth v2 and KITTI on other datasets containing both indoor and outdoor scenes, without any fine-tuning. For SUN RGBD dataset, due to the different resolutions of the images, we centrally cropped all images to 512 × 384. For DIODE and ETH-3D datasets, the resolutions of the images are 1024 × 768 and 6048 × 4032, respectively. We bilinearly resampled the images to 512 × 384 and 640 × 320, respectively. For ScanNet dataset, we centrally cropped the images with the resolution of 1296 × 968 to 1280 × 960. The maximum evaluation mask is 10 m in indoor scenes and 80 m in outdoor scenes. For evaluation of all outputs, the central cropping scheme proposed by Grag et al.^27^ is adopted. The quantitative comparison of model generalization ability in indoor and outdoor scenes are shown in Tables 4 and 5, respectively. Note that all methods show poor cross-dataset generalization performance, but the method proposed by Ranftl et al.^3^ benefits from the use of additional datasets for training, and its performance is relatively better than others.

**Table 4:** Quantitative comparison of model generalization ability in indoor scenes.

Method	SUN RGBD		DIODE indoor		ETH-3D indoor		ScanNet	
	δ_1_↑	REL↓	δ_1_↑	REL↓	δ_1_↑	REL↓	δ_1_↑	REL↓
Lee et al.^9^	0.448	0.528	0.307	0.380	0.332	0.424	0.267	0.367
Bhat et al.^14^	*0.553*	*0.334*	0.265	0.407	*0.348*	0.375	0.643	0.208
Ranftl et al.^3^	0.500	0.355	**0.439**	**0.323**	**0.465**	**0.294**	**0.744**	**0.190**
Proposed	**0.568**	**0.328**	*0.361*	*0.352*	0.343	*0.371*	*0.695*	*0.193*

The best results are shown in bold, and the second best results are italics.

**Table 5:** Quantitative comparison of model generalization ability in outdoor scenes.

Method	DIODE outdoor				ETH-3D outdoor			
	δ_1_↑	δ_3_↑	REL↓	RMS↓	δ_1_↑	δ_3_↑	REL↓	RMS↓
Lee et al.^9^	*0.196*	*0.551*	*0.656*	*10.071*	*0.208*	*0.546*	0.904	*5.472*
Bhat et al.^14^	0.169	0.534	0.679	10.184	0.127	0.515	*0.886*	5.872
Ranftl et al.^3^	**0.247**	**0.761**	**0.647**	**7.492**	**0.261**	0.519	0.991	**5.147**
Proposed	0.174	0.512	0.699	10.453	0.123	**0.560**	**0.819**	5.659

The best results are shown in bold, and the second best results are italics.

**Figure 3:**
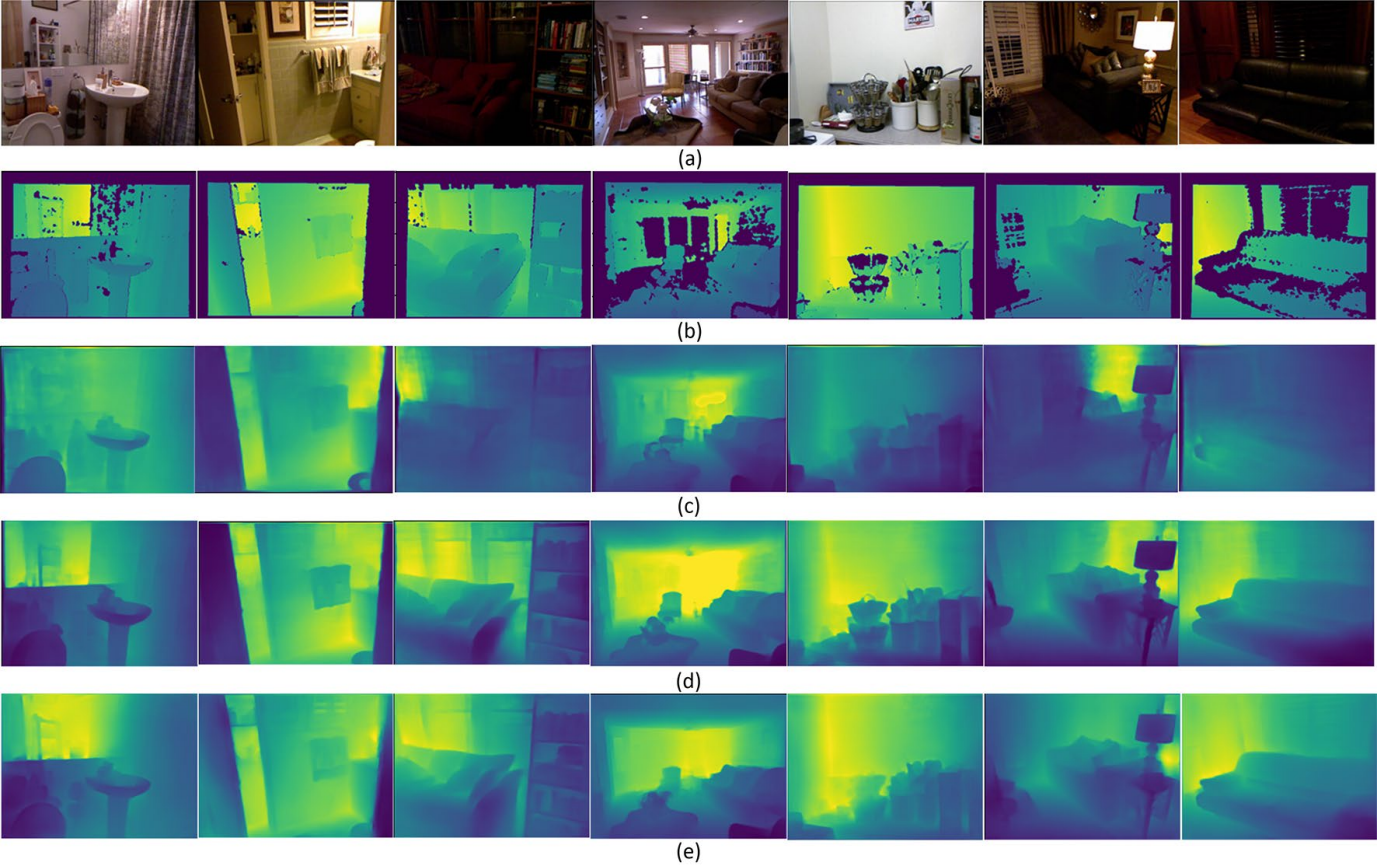
Qualitative evaluation of depth maps on NYU Depth v2 dataset. (**a**) input RGB images; (**b**) ground-truth depth maps; depth maps predicted by (**c**) Bhat et al.^14^, (**d**) Ranftl et al.^3^, and (**e**) proposed model.

**Figure 4:**
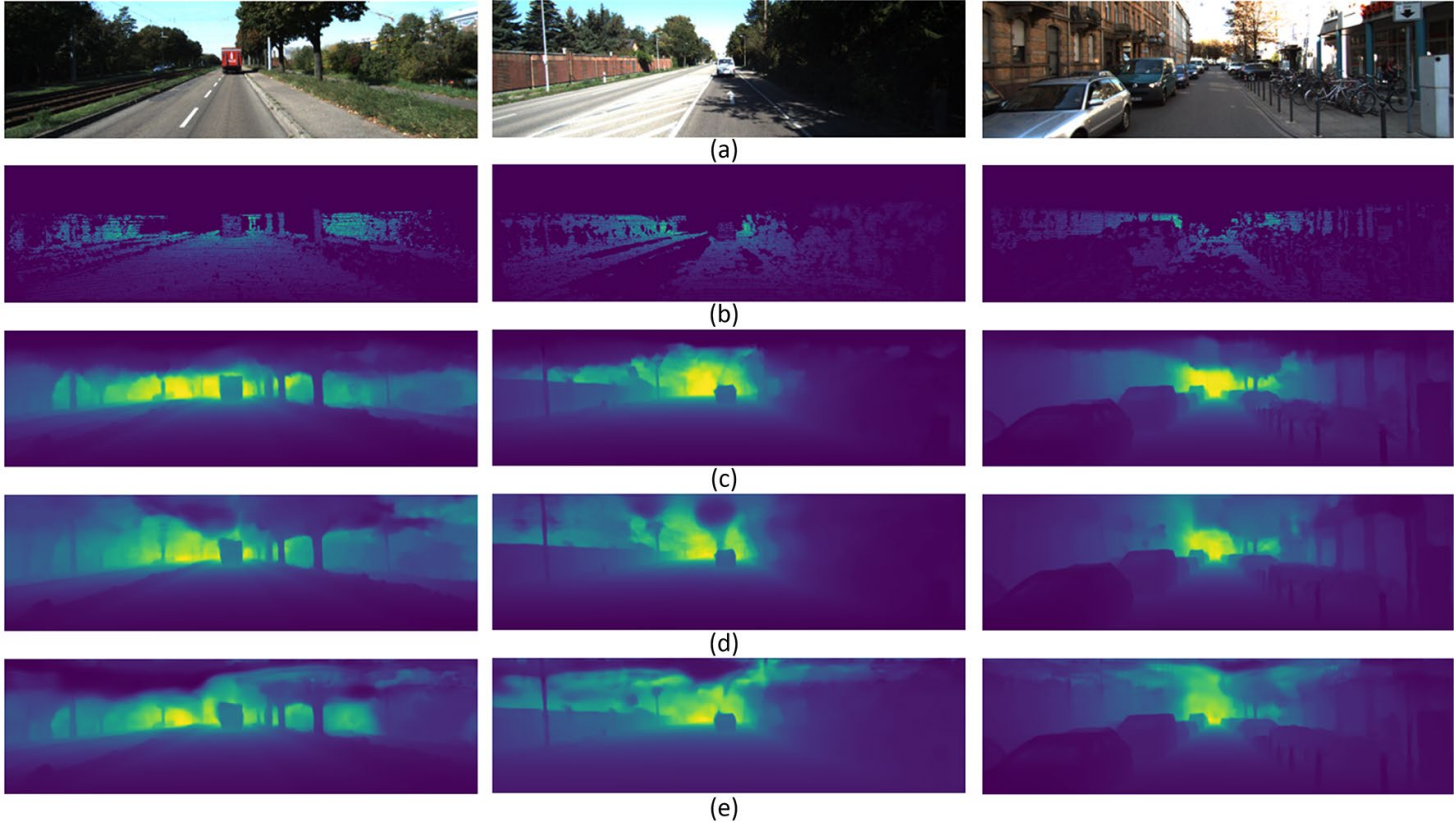
Qualitative evaluation of depth maps on KITTI dataset. (**a**) input RGB images; (**b**) ground-truth depth maps; depth maps predicted by (**c**) Bhat et al.^14^, (**d**) Ranftl et al.^3^, and (**e**) proposed model.

*Qualitative evaluation*. Figures 3 and 4 show the depth prediction results of the proposed model for a single RGB image in certain scenarios in NYU Depth v2 and KITTI datasets, respectively, and compare them with the two state-of-the-art prediction results provided in^14^ and^3^. As shown in the figure, the proposed model can restore depth information from 2D images well, with results comparable to the state-of-the-art methods in most scenarios and better in some scenarios.

**Figure 5:**
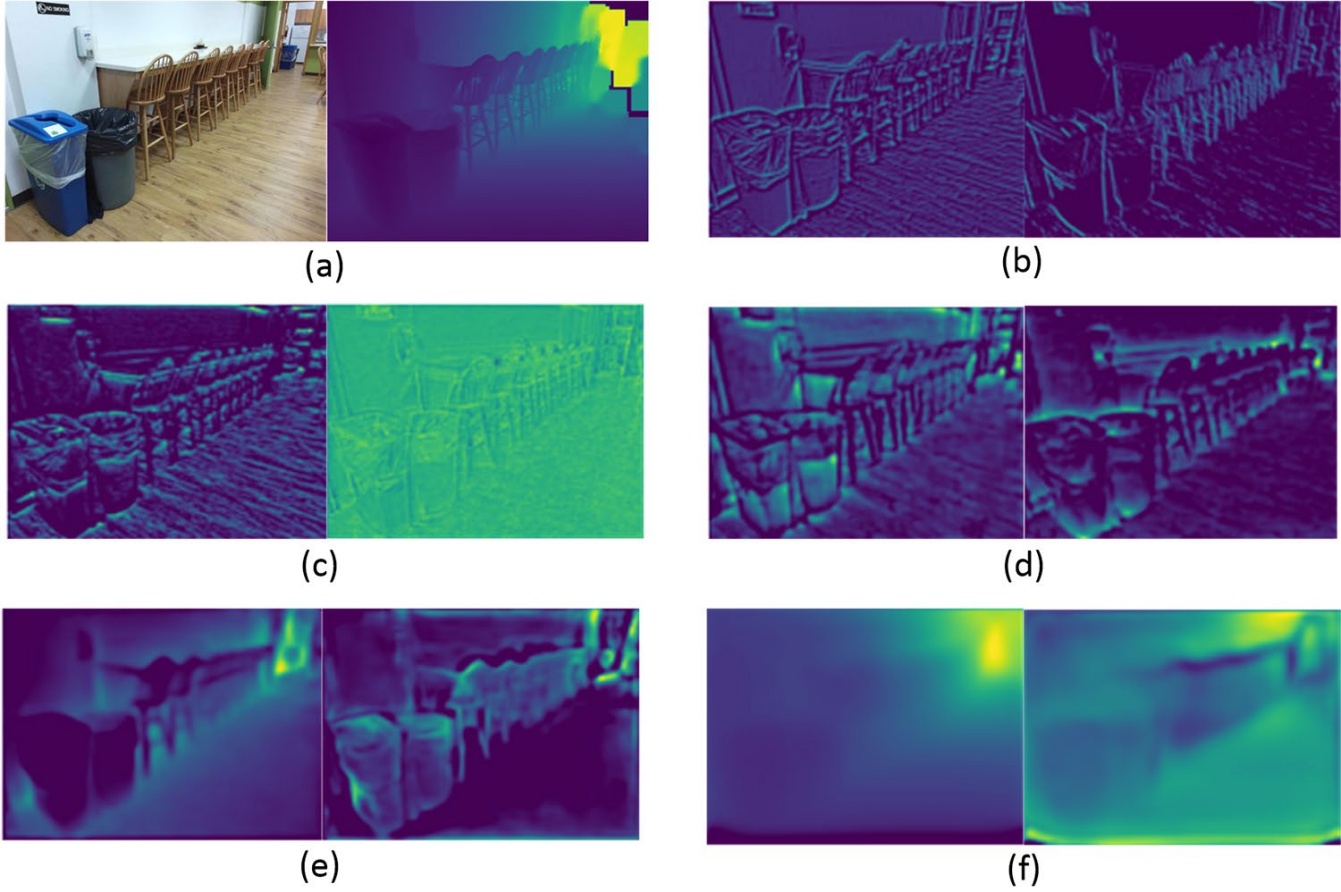
Visualization of decoded features. (**a**) RGB image and the ground truth. (**b**) Several channels in (**b**) *features1*, (**c**) *features2*, (**d**) *features3*, (**e**) *features4*, and (**f**) *features5*.

We further evaluated the proposed method by visualizing the decoded features as shown in Fig. 5. Obviously, after decoding the shallower features of the encoder in the proposed network architecture, the feature maps contain a significant amount of detailed texture and spatial information. With the deepening of the network, the decoded feature map can reflect the depth information globally.

**Figure 6:**
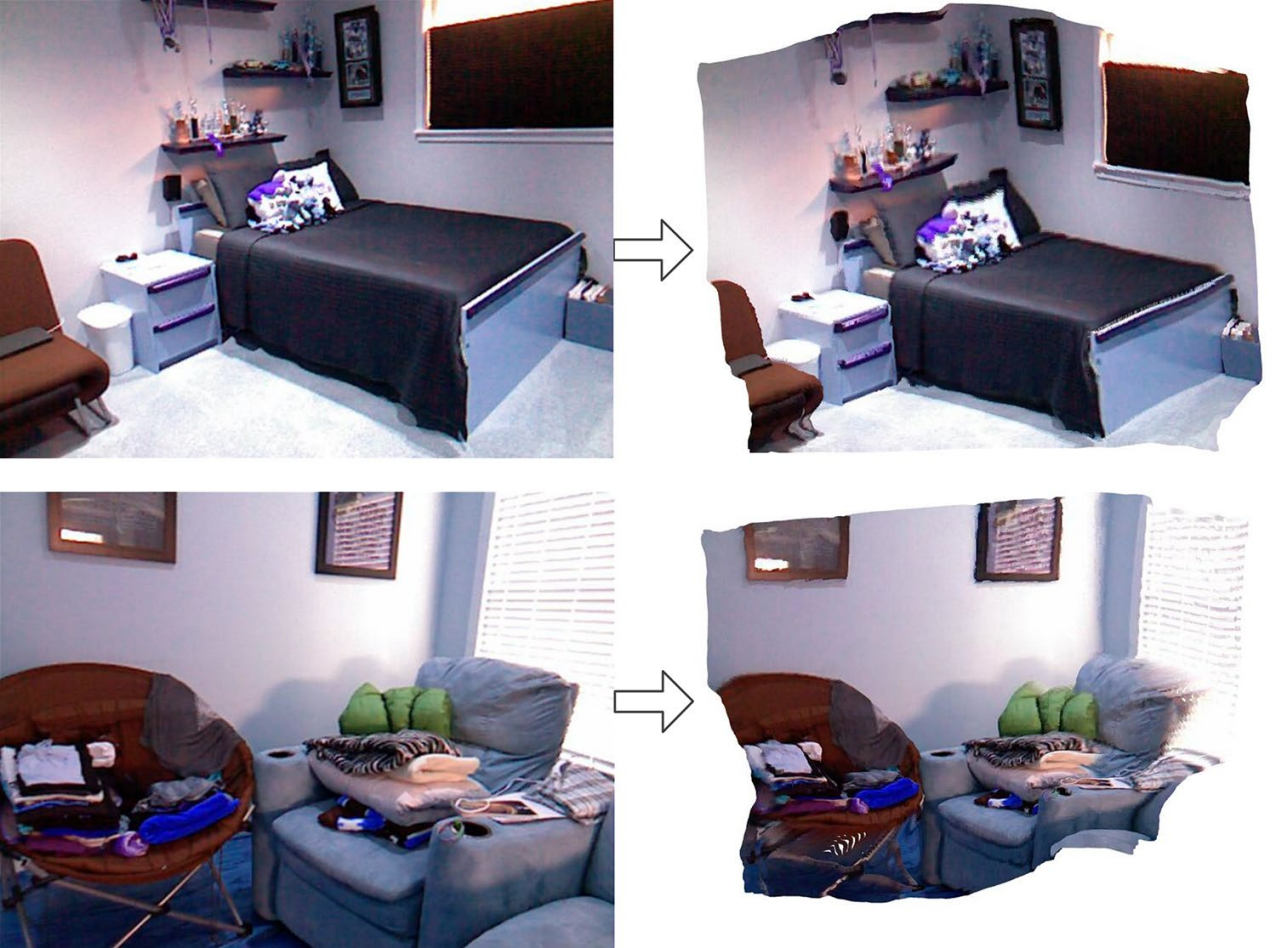
Depth predicted by the proposed model generates a 3D point cloud result.

Converting a 2D image into a 3D point cloud is an important application of depth estimation. We therefore also evaluated our method by evaluating the quality of the 3D point cloud generated by the depth map. Figure 6 shows the 3D point cloud generated by the proposed method after predicting the depth of some scenes. The generated 3D point cloud has good quality and can reflect the actual 3D space in general.

### Ablation study

We performed several ablation studies to analyze several factors that may have an impact on our results. In these studies, except for the different subjects studied, the other strategies were the same in each group of experiments. The factors analyzed were the following.

*Parallel prediction decoder*. We first evaluated the effectiveness of the proposed decoder, which implements parallel prediction of global and local depth information. The structure of the comparison method is similar to^8^ and the backbone structure of^14^. Specifically, it upsamples the features of the deepest layer of the encoder to the same resolution as the features of the sub-deep layer, and then concatenates the features after resampling with the features of the sub-deep layer so as to gradually reach the shallowest layer. We trained 20 epochs on NYU Depth v2 dataset, and the quantitative comparison results are shown in Table 6. It can be seen from the table that the proposed parallel method has advantages over the serial method in terms of accuracy, error, and parameter size.

**Table 6:** Quantitative comparison of serial and parallel decoders.

Method	δ_1_↑	δ_2_↑	δ_3_↑	REL↓	RMS↓	Log_10_↓	Param↓
Serial	0.897	0.983	**0.996**	**0.102**	0.367	0.045	109 M
Parallel	**0.905**	**0.984**	**0.996**	**0.102**	**0.362**	**0.043**	**77 M**

The better results are shown in bold.

**Figure 7:**
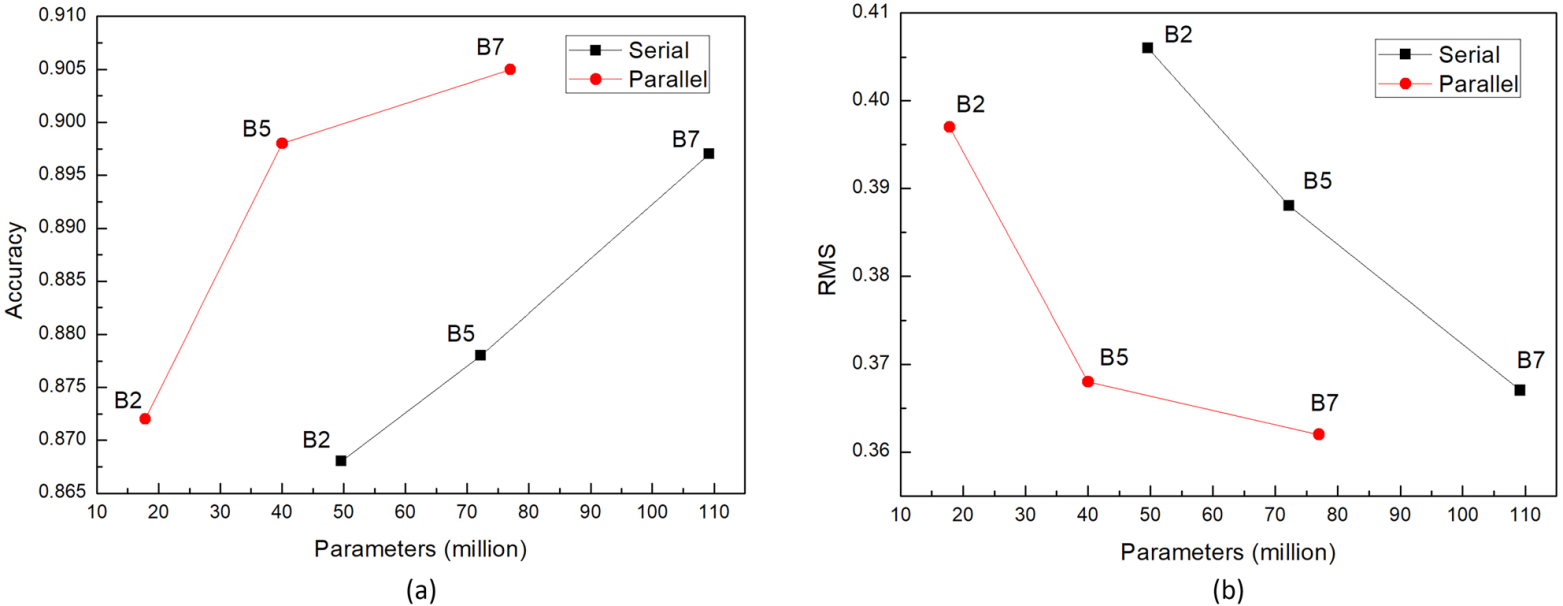
Performance comparisons of (**a**) accuracy and (**b**) error of serial and parallel decoders using different encoders.

Moreover, we replaced different encoders to further study the boosting effect of the proposed parallel decoder. After changing the encoder from Efficientnet-B7 to B2 and B5, we separately used the traditional and proposed decoders for training, and the results are shown in Fig. 7. We recorded the accuracy (δ_1_) and error (RMS) of the training results separately and the comparison of the model parameters is reflected in the figure. The figure demonstrates that the proposed parallel decoder is a significant improvement over the traditional serial decoder.

*Transformer for fusing parallel prediction*. We introduced an experiment to study the effectiveness of using Transformer for fusion in the proposed decoder. The calculation method for comparison is

12 $$output = \mathop \sum \limits_{i = 1}^{4} \left( {{\text{F}}_{sigmoid} \left( {{\text{F}}_{conv} \left( {blocki} \right)} \right) \times blocki} \right),$$

wherein the *blocki* (1 ≤ *i* ≤ 4) is first convolved, and then the sigmoid function is used to map it to between 0 and 1 and then multiply with the *blocki*; finally, all the *blocki* are added pixel by pixel to obtain the output. Note that the encoder in this experiment used EfficientNet-B3 pre-trained on ImageNet, which has fewer parameters and is faster to train. We trained 20 epochs on NYU Depth v2 dataset, and the quantitative comparison results are shown in Table 7. It can be seen from the table that using Transformer has advantages in terms of accuracy and error compared with simple calculation fusion, and the parameters do not increase too much.

**Table 7:** Quantitative comparison of different fusion methods.

Method	δ_1_↑	δ_2_↑	δ_3_↑	REL↓	RMS↓	Log_10_↓	Param↓
Sigmoid	0.867	0.978	**0.996**	0.117	0.401	0.049	**21.02 M**
Transformer	**0.877**	**0.979**	0.995	**0.112**	**0.398**	**0.048**	21.14 M

The better results are shown in bold.

*Number of depth intervals*. We then introduced a set of experiments to study the influence of the number of depth intervals *b* in the pixel-level depth loss for *block1* to *block4* on the experimental results. We mainly conducted three experiments, with depth interval numbers of 1, 4, and 10. Note that the encoder in this set of experiments also used EfficientNet-B3 pre-trained on ImageNet. We trained 20 epochs on NYU Depth v2 dataset, and the quantitative comparison results are shown in Table 8. The table shows that the number of depth intervals may have little impact on the experimental results, and we thus chose a relatively better 10-interval division in the loss item.

**Table 8:** Quantitative comparison of different numbers of depth intervals.

Intervals	δ_1_↑	δ_2_↑	δ_3_↑	REL↓	RMS↓	Log_10_↓
1	0.875	0.979	**0.996**	0.115	**0.392**	0.052
4	0.872	**0.980**	**0.996**	0.113	0.400	0.049
10	**0.877**	0.979	0.995	**0.112**	0.398	**0.048**

The better results are shown in bold.

*Loss function for pixel-level depth of final prediction*. To study the effect of the combination of variance and mean-squared error used in the pixel-level loss in the final prediction, we compared the loss term with the scale invariant (SI) loss introduced by Eigen et al.^1^. We trained 20 epochs on NYU Depth v2 dataset, and the quantitative comparison results are shown in Table 9. It can be seen from the table that, except for ${\delta }_{1}$, the values on the other indicators are the same. Therefore, it can also be seen that using the loss constraint we proposed can better control the small, difficult-to-predict parts of the scene.

**Table 9:** Quantitative comparison of different loss functions for pixel-level depth of final prediction.

Loss	δ_1_↑	δ_2_↑	δ_3_↑	REL↓	RMS↓	Log_10_↓
SI	0.898	**0.984**	**0.996**	**0.102**	**0.362**	**0.043**
Ours	**0.905**	**0.984**	**0.996**	**0.102**	**0.362**	**0.043**

The better results are shown in bold.

*Loss function for pixel-level depth loss for block1 to block4.* To study the effectiveness of the loss term that we designed for *block1* to *block4*, we removed the loss term and conducted an experiment. We trained 20 epochs on NYU Depth v2 dataset, and the quantitative comparison results are shown in Table 10. It can be seen from the table that using this loss term during training can obtain better results.

**Table 10:** Quantitative comparison of different loss functions for pixel-level depth of *block1* to *block4*.

Method	δ_1_↑	δ_2_↑	δ_3_↑	REL↓	RMS↓	Log_10_↓
Without loss term	0.895	**0.985**	**0.997**	0.105	0.366	0.044
With loss term	**0.905**	0.984	0.996	**0.102**	**0.362**	**0.043**

The better results are shown in bold.

**Figure 8:**
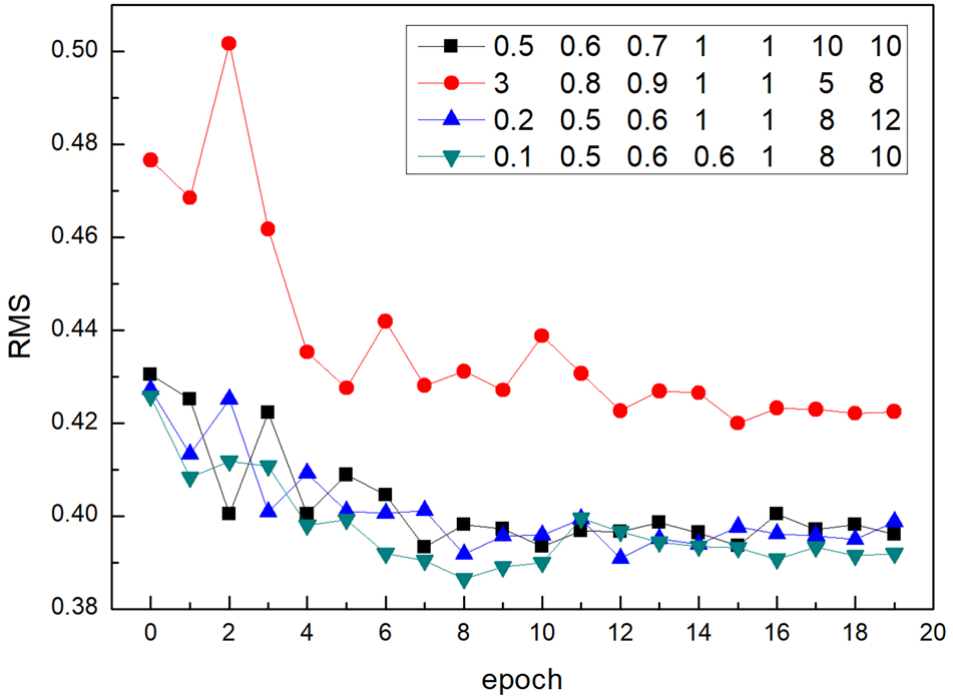
Comparison of the effect of using different loss function weights on training.

*Weight of each loss term*. The weight of the loss item will directly affect the model training process and final result. To roughly find a suitable set of weight combinations, we conducted ablation studies with different values for ${\lambda }_{1}, { \lambda }_{2}, {\lambda }_{3},$ and ${\lambda }_{4}$ in Eq. (2) and $\eta , \alpha ,$ and $\beta$ in Eq. (5). We trained 20 epochs on NYU Depth v2 dataset and recorded the RMS error as shown in Fig. 8, where the numbers in the legend, reading across, represent the values of $\eta , {\lambda }_{1}, { \lambda }_{2}, {\lambda }_{3}, {\lambda }_{4}, \alpha ,$ and $\beta$, respectively. It can be seen that, when $\eta =0.1$, ${\lambda }_{1}=0.5$, ${\lambda }_{2}={\lambda }_{3}=0.6$, ${\lambda }_{4}=1$, $\alpha =8$, and $\beta =10$, the error that the model can achieve after training is the lowest.

## Conclusions

In this work, we propose a novel end-to-end supervised monocular depth estimation network and achieve state-of-the-art results. We design a decoder that realizes parallel prediction and then fuses the predictions for output. We deploy this decoder into multiple stages of an encoder, combine a loss term based on depth interval partitioning to obtain significant improvements, and present a number of experimental results on challenging benchmarks to validate it. In future work, we aim to apply this work to specific applications, such as 3D reconstruction.

## Data Availability

All data generated or analyzed during this study are included in this published article. We provide the proposed model code and pre-training parameters at: https://github.com/jt-liu/PDNet, accessed on 25 September 2022.
